# Analysis of *Phakopsora pachyrhizi* transcript abundance in critical pathways at four time-points during infection of a susceptible soybean cultivar using deep sequencing

**DOI:** 10.1186/1471-2164-14-614

**Published:** 2013-09-11

**Authors:** Arianne Tremblay, Parsa Hosseini, Shuxian Li, Nadim W Alkharouf, Benjamin F Matthews

**Affiliations:** 1 Soybean Genomics & Improvement Laboratory, United States Department of Agriculture-Agricultural Research Service (USDA-ARS), Beltsville, MD 20705, USA; 2 Bioinformatics/Computational Biology, George Mason University, 4400 University Dr. Manassas, Fairfax, VA 22030, USA; 3 Computational Biology Branch, National Center for Biotechnology Information, National Institutes of Health, Bethesda, MD, USA; 4 USDA-ARS, Crop Genetics Research Unit, Stoneville, MS 38776, USA; 5 Molecular Biology, Biochemistry and Bioinformatics, Fischer College of Science and Mathematics, Towson University, 8000 York Road, Towson, MD 21252, USA; 6 Department of Biological Sciences, University of Maryland Baltimore County, 1000 Hilltop Circle, BS411/412, Baltimore, MD 21250, USA

**Keywords:** Deep sequencing, Transcript abundance, *Phakopsora pachyrhizi*, Plant-pathogen interaction, Soybean, Soybean rust

## Abstract

**Background:**

*Phakopsora pachyrhizi*, the causal agent responsible for soybean rust, is among the top hundred most virulent plant pathogens and can cause soybean yield losses of up to 80% when appropriate conditions are met. We used mRNA-Seq by Illumina to analyze pathogen transcript abundance at 15 seconds (s), 7 hours (h), 48 h, and 10 days (d) after inoculation (ai) of susceptible soybean leaves with *P. pachyrhizi* to gain new insights into transcript abundance in soybean and the pathogen at specific time-points during the infection including the uredinial stage.

**Results:**

Over three million five hundred thousand sequences were obtained for each time-point. Energy, nucleotide metabolism, and protein synthesis are major priorities for the fungus during infection and development as indicated by our transcript abundance studies. At all time-points, energy production is a necessity for *P. pachyrhizi,* as indicated by expression of many transcripts encoding enzymes involved in oxidative phosphorylation and carbohydrate metabolism (glycolysis, glyoxylate and dicarboxylate, pentose phosphate, pyruvate). However, at 15 sai, transcripts encoding enzymes involved in ATP production were highly abundant in order to provide enough energy for the spore to germinate, as observed by the expression of many transcripts encoding proteins involved in electron transport. At this early time-point, transcripts encoding proteins involved in RNA synthesis were also highly abundant, more so than transcripts encoding genes involved in DNA and protein synthesis. At 7 hai, shortly after germination during tube elongation and penetration, transcripts encoding enzymes involved in deoxyribonucleotide and DNA synthesis were highly abundant. At 48 hai, transcripts encoding enzymes involved in amino acid metabolism were highly abundant to provide for increased protein synthesis during haustoria maturation. During sporulation at 10 dai, the fungus still required carbohydrate metabolism, but there also was increased expression of transcripts encoding enzymes involved in fatty acid metabolism.

**Conclusion:**

This information provides insight into molecular events and their timing throughout the life cycle of the *P. pachyrhizi*, and it may be useful in the development of new methods of broadening resistance of soybean to soybean rust.

## Background

*Phakopsora pachyrhizi*, the causal agent of soybean rust (SR), is among the top hundred most virulent plant pathogens. *P. pachyrhizi* can infect at least 89 different plants from bean to lupine. In the United States, soybean (*Glycine max*) is the only crop for which a yield effect has been reported from *P. pachyrhizi* infection. Infection has been reported on other crops in the U.S., but these infections are of limited scope with no apparent economic impact. These crops include scarlet runner bean, lima bean, and kidney bean. Kudzu and beggarweed have also been identified in the U.S. as non-crop host plants [1-4].

In Asia, where the pathogen originated, soybean yield losses range from 20% to 80% following *P. pachyrhizi* infection [5,6]. Soybean in South America is also highly affected by SR. Since 2001, when the disease was first observed in Paraguay [7], soybean yield losses of 10% to 50% have been quite common [8]. The SR disease monitoring program established in the U.S. allows farmers to respond quickly to outbreaks of SR infection by applying fungicides when and where SR disease develops to avoid yield losses. Although fungicide application decreases *P. pachyrhizi* development on soybean crops, the spread of the disease throughout the US has still increased over the past several years. In 2009, Mississippi recorded for the first time a yield reduction associated with SR disease, of between 8% and 25% [9]. Since 2010, the precarious state of funding of the U.S. monitoring program has provided impetus to researchers to understand the interaction between *P. pachyrhizi* and its soybean host and to identify new forms of resistance to this pathogen in soybean. Most of the efforts to develop new resistance in commercial soybean cultivars have been concentrated on the identification of soybean genes from six major loci (*Rpp*1-*Rpp*6 and *Rpp?*) conferring resistance to *P. pachyrhizi*[10-13]. The specific gene responsible for SR resistance mediated by the *Rpp*4 locus was identified by Meyer et al. [14] as encoding a gene belonging to the CC-NBS-LRR family of disease resistance genes showing greatest similarity to the RGC2 family of disease resistance genes from lettuce.

*P. pachyrhizi* is an obligate biotroph which makes it hard to study as a unique entity. Fungal spores are spread by air currents and once they land on a leaf surface and optimal growth conditions are reached, they begin to germinate and form appressoria. This first stage of rust infection, named the pre-penetration stage, happens within 24 h of a spore landing on the leaf. This is the only infection stage occurring outside the host. The germination step can be reproduced in water. Posada-Buitrago and Frederick [15] and Stone et al. [16] isolated mRNA from germinating *P. pachyrhizi* urediniospores and appressoria, constructed cDNA libraries, and sequenced 488 and 1,029 unique expressed sequence tags (ESTs) respectively available at NCBI. Since then more *P. pachyrhizi* ESTs from this specific physiological stage have been added to the NCBI database as well as ESTs from germinating spores of other plant pathogenic fungi such as *Puccinia striiformis* f. sp. *tritici*[17], *P. triticina*[18], *Ustilago maydis*[19] and *Fusarium oxysporum*[20]. Following the pre-penetration stage up to 48 hours after a spore lands, infection hyphae form that allow direct penetration inside the host and subsequent formation of primary haustoria mother cells (HMCs). This second step in infection is named the penetration stage.

From the formation of HMCs to eight days after spore landing, fungal intercellular hyphae grow between host palisade and mesophyll cells, more haustoria cells are produced, and uredinia arise as a result of hyphae aggregation. During this colonization stage, haustorial cells can be isolated using a protocol established by Hahn and Mendgen [21]. Loehrer and Schaffrath [22] isolated *P. pachyrhizi* haustoria, extracted RNA, constructed a cDNA library, and sequenced the whole haustorial transcriptome, resulting in 111,440 *de-novo* assembled contigs. Transcript abundance in haustorial cells has also been studied in few other rusts including *P. striiformis* f. sp. *tritici*, *Uromyces appendiculatus*, *U. fabae,* and *Melampsora lini*[23-26].

Fewer studies have been conducted on transcript abundance within hyphae since they are hard to isolate from plant material. However, more transcript abundance studies have been done on cDNA libraries constructed from mRNA extracted from whole infected leaves at different time-points along the infection process. There are 5,981 ESTs from soybean cultivar Williams 82 at 6 to 8 days after inoculation (dai) with urediniospores of *P. pachyrhizi* isolate Taiwan 72–1 (TW72-1) and 6,390 ESTs from soybean cultivar Williams 82 at 13 to 15 dai with urediniospores of *P. pachyrhizi* isolate TW72-1 deposited in the NCBI database (Posada-Buitrago et al., 2006; unpublished data). Although the majority of these ESTs correspond to soybean genes, some correspond to *P. pachyrhizi* genes.

Between nine and ten days after urediniospores land on the leaf surface, new urediniospores are produced inside the uredinium. This represents the sporulation stage. Urediniospore production can extend up to three weeks from the generation of the first uredinium, but secondary uredinia can maintain sporulation for up to 15 weeks [27]. During the sporulation stage, the plant cuticle is ruptured and urediniospores are released into the environment. Transcript abundance analyses were performed on urediniospores of *P. pachyrhizi* collected on soybean infected leaves by Posada-Buitrago et al. in 2006 (unpublished), resulting in 2,122 ESTs being deposited in the NCBI database. Similar analyses were performed on spores of *P. striifromis* f. sp. *tritici*[28].

Although obligate biotrophic organisms are difficult to study during the infection process, laser capture microdissection (LCM) can help to circumvent this difficulty by enabling the dissection of infected tissue precisely enough to isolate the organism within the host. Our research team used LCM to isolate uredinia of *P. pachyrhizi* formed 10 dai on leaves of the susceptible soybean cultivar Williams 82, and we analyzed transcript abundance at this specific stage of *P. pachyrhizi* development [28]. Sanger sequencing of our library allowed us to generate a limited number of ESTs. More recently, Hacquard et al. [29] used LCM to isolate uredinial sites corresponding to spores and sporogenous hyphae, fungal infection tissues in spongy mesophyll, and fungal infection tissues in palisade mesophyll from poplar leaves 4 and 7 dai with *Melamspora larici-populina*. RNA was extracted and amplified from the cells isolated by LCM and the RNA was hybridized to whole-genome exon oligoarrays of *M. larici-populina*. Even though array technology provides the means to study the expression of many genes, it does not allow the discovery of new genes. Based on these limitations, we deep-sequenced cDNA libraries of soybean cultivar Williams 82 inoculated with *P. pachyrhizi* along a time-course of infection using the Illumina platform to generate a more in-depth transcript abundance profile of the genes expressed by soybean and *P. pachyrhizi* simultaneously. One objective of this study was to identify *P. pachyrhizi* abundant transcripts present at different times during an infection process, build a model of what is happening during the infection process based on transcript abundance and, choose target genes for silencing or overexpression in order to produce a resistant plant. This information will help us to better understand the infection process of *P. pachyrhizi* on soybean plants and provide new ways to engineer soybean plants to fight the pathogen attack. This manuscript described transcript abundance profile of genes expressed by *P. pachyrhizi* and provides insights into the uredinial stage and infection process of *P. pachyrhizi* at the molecular level. We show that genes encoding enzymes involved in specific biochemical pathways are expressed at specific time points to help meet the requirements of *P. pachyrhizi* to successfully parasitize soybean and produce urediniospores.

## Results and discussion

### mRNA sequencing of soybean infected with *Phakopsora pachyrhizi*

Four time-points were chosen for this study, namely fifteen seconds after inoculation (15 sai), seven hours after inoculation (7 hai), 48 hai, and 10 days after inoculation (dai) representing pre-penetration, colonization, and sporulation stages. Since we are interested in identifying *P. pachyrhizi* transcripts abundant during the infection process to engineer a strategy of host resistance, knowing which genes are expressed as the earliest infection stage may be key. For this reason, we chose 15 sai and 7 hai when urediniospores get in contact with their soybean host and begin to germinate. Stopping these steps by silencing a gene highly expressed or over-expressing a down-regulated gene during this specific stage may be a good strategy. Haustoria are infection structures playing many important roles during the infection process. Thus, knowing which genes are expressed at 48 hai corresponding to the colonization stage where haustoria develop is crucial to understanding the process of pathogen infection. Even though the sporulation stage occurs late in the infection process (10 dai), it is still important, because slowing down or preventing urediniospores formation will reduce secondary infection on plants and spread to other fields. For all these reasons, 15 sai, 7 hai, 48 hai and 10 dai were chosen, RNA was isolated from soybean trifoliate leaves inoculated with *P. pachyrhizi* at these specific time-points, and cDNA was sequenced. Between 3,510,311 and 9,082,363 reads per lane was obtained. Table 1 described reads information associated with each time-point.

**Table 1:** **Summary of sequenced reads throughout a soybean-*P. pachyrhizi *infection study**

**Time-points**	**SRA accession**	**# Raw reads**	**# QA/QC**^**a **^**reads after all filtering**	**# De-novo transcripts**
15 sai	SRR445529	4,467,871	1,220,368	6531
7 hai	SRR610280	7,543,421	2,997,508	4627
48 hai	SRR610284	9,082,363	2,124,743	4273
10 dai	SRR445528	3,510,311	1,947,057	12284

^**a**^Quality Assurance/Quality Control.

Reads not aligning to the soybean genome were separated from reads aligning to the soybean genome and analyzed as potential *P. pachyrhizi* sequences. Between 23 and 55 percent of the total reads per lane were considered as potential *P. pachyrhizi* sequences; sequences which were then assembled to build *de-novo* transcripts (Table 1)*.* Even though our analysis has been focused on contigs of 75 bp and longer, some additional contigs shorter than 75 bp have been included based on their importance in different metabolic pathways and developmental processes. Table 2 listed some examples of these contigs.

**Table 2:** Contigs of importance, shorter than 75 bp, taking into account in our analysis

**Contig length**	**Similarity search results**	**E.C. number**	**k-mer coverage**	**Normalized coverage**
70 bp	NADH dehydrogenase subunit f	1.6.5.3	1.54	2.450
62 bp	Fructose-1,6-bisphosphatase	3.1.3.11	1.62	2.790
72 bp	Pectin methylesterase 1	N.A.^a^	1.58	2.473
69 bp	Penicillin amidase	3.5.1.11	1.71	2.744
64 bp	Polyphosphate kinase	2.7.4.1	1.26	2.122
59 bp	Xanthine dehydrogenase	1.17.1.4	1.35	2.414
50 bp	Gamma-glutamyl phosphate reductase	N.A.	1.78	3.708
55 bp	Methylenetetrahydrofolate reductase	N.A.	1.72	3.262
73 bp	Pyrophosphate-dependent phosphofructokinase	2.7.1.11	1.56	2.423
52 bp	Urease	3.5.1.5	1.09	2.180
68 bp	Glucokinase	2.7.1.2	1.47	2.380
58 bp	Malate dehydrogenase	1.1.1.37	1.96	3.553
59 bp	Phosphoribosylformylglycinamidine cyclo-ligase	6.3.3.1	1.52	2.718
54 bp	Phosphoribosylaminoimidazole carboxylase	4.1.1.21	2.14	4.127
56 bp	Pyruvate dehydrogenase (acetyl-transferring)	1.2.4.1	1.57	2.931
51 bp	2,3-bisphosphoglycerate-independent phosphoglycerate mutase	5.4.2.1	1.11	2.264
65 bp	NAD-specific glutamate dehydrogenase	1.4.1.2	1.81	3.017

^a^Not applicable since no E.C. number has been allocated to these proteins.

As previously mentioned, sequence information from *P. pachyrhizi* is limited and mostly derived from cDNA libraries constructed from mRNA extracted from urediniospores and germinated urediniospores as well as from whole infected soybean leaves from 6 to 15 dai. By using a deep sequencing strategy, we sequenced and generated 27,715 contigs from four time-points which represent a little more than half of what is available at NCBI for *P. pachyrhizi* (49,596 ESTs). By using mRNA-Seq, we also obtained sequences from additional infection stages including 15 sai, 7 hai and 48 hai which were not yet covered at NCBI public databases. From the present data set only 8,673 contigs shared similarity to *P. pachyrhizi* ESTs previously identified. Thus, more than 19,000 contigs, more than two-thirds of the total number of contigs, represent newly identified *P. pachyrhizi* transcripts. Using genomic, transcriptomic, proteomic, and metabolomic information available from other rusts and pathogenic fungi, we interpreted our deep sequencing data in relation to the needs and requirements of *P. pachyrhizi* during infection of susceptible soybean leaves.

### Genes encoding enzymes involved in energy production and carbohydrate metabolism were abundant at all time-points

The number of DNA sequence reads building each contig was used to determine amount of expression of each gene. This was used to compare transcript abundance among all time-points. There was an increase in the number of different transcripts identified from 15 sai to 10 dai, suggesting that as the fungus grows, spreads, and matures, it expresses more genes to complete its infection. At 48 hai and 10 dai, approximately 70% and 90% of the transcripts encoding proteins sharing similarity to known proteins identified in different databases were up-regulated while at 7 hai only approximately 30% of the transcripts encoding proteins sharing similarity to known proteins identified in different databases were up-regulated.

**Figure 1:**
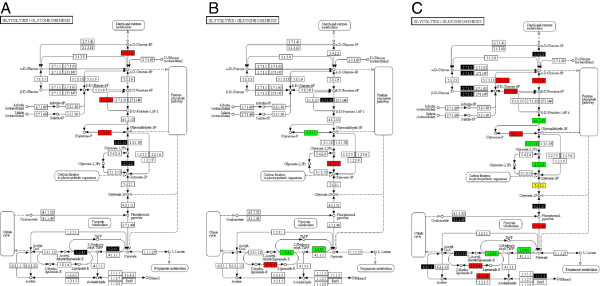
**Representation of the glycolysis pathway in which regulation status of transcripts encoding enzymes identified at (A) 7 hai, compared to 15 sai, (B) 48 hai, compared to 7 hai, and (C) 10 dai, compared to 48 hai, is annotated.** Boxes colored in red represent enzymes encoded by down-regulated transcripts; boxes colored in green represent enzymes encoded by up-regulated transcripts; while boxes colored in yellow represent enzymes encoded by transcripts with varied regulatory status. Boxes colored in black represent enzymes encoded by transcripts identified at the experimental time-point but not at the previous time point.

Forty transcripts encoding proteins sharing similarity to known proteins identified in different databases were common to all time-points. The expression of approximately half of these transcripts encoding proteins declined at 7 hai compared to 15 sai, followed by an impressive increase at 48 hai of the expression levels of all except three of these transcripts encoding proteins. At 10 dai, the expression of approximately two thirds of these transcripts decreased while the expression of one third still increased. For example, the expression of genes encoding certain enzymes in gluconeogenesis suggests that the pathway direction where β-D-glucose 1,6-bisphosphate is converted in β-D-glucose 1,6-phosphate by fructose-1,6-phosphatase I (3.1.3.11) changed at 15 sai, either for energy storage or for providing glucose-6-phosphate for the pentose phosphate pathway for NADH production. However, the expression of these genes encoding enzymes involved in this pathway was down-regulated at 7 hai in favor of genes encoding proteins involved in pyruvate production (Figure 1A). Changes in transcript abundance were also observed in other metabolic processes between 15 sai and 7 hai such as fructose and mannose metabolism, pentose phosphate pathway and nitrogen metabolism. Further investigations are needed to understand why these changes occur between induction of germination and 7 hai.

**Figure 2:**
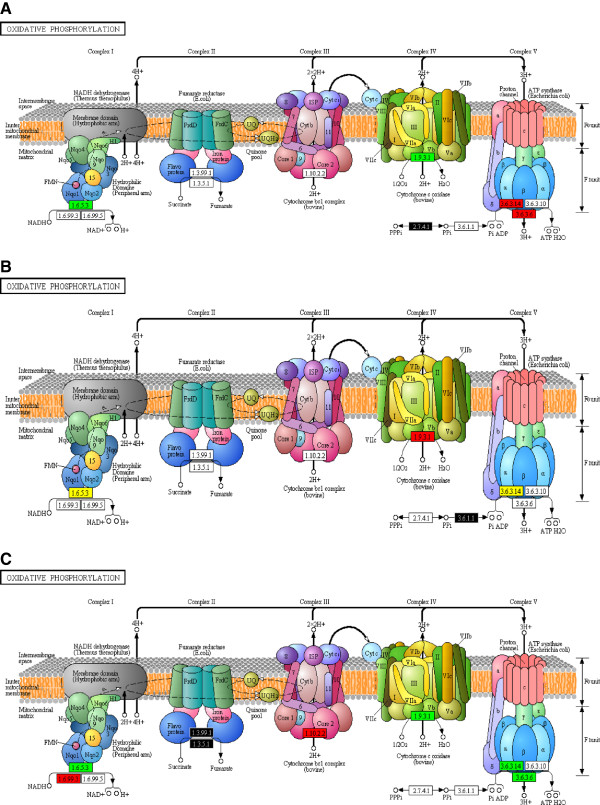
**Representation of the oxidative phosphorylation pathway in which regulation status of transcripts encoding enzymes identified at (A) 7 hai, compared to 15 sai, (B) 48 hai, compared to 7 hai, and (C) 10 dai, compared to 48 hai, is annotated.** Color coding as in Figure 1.

**Figure 3:**
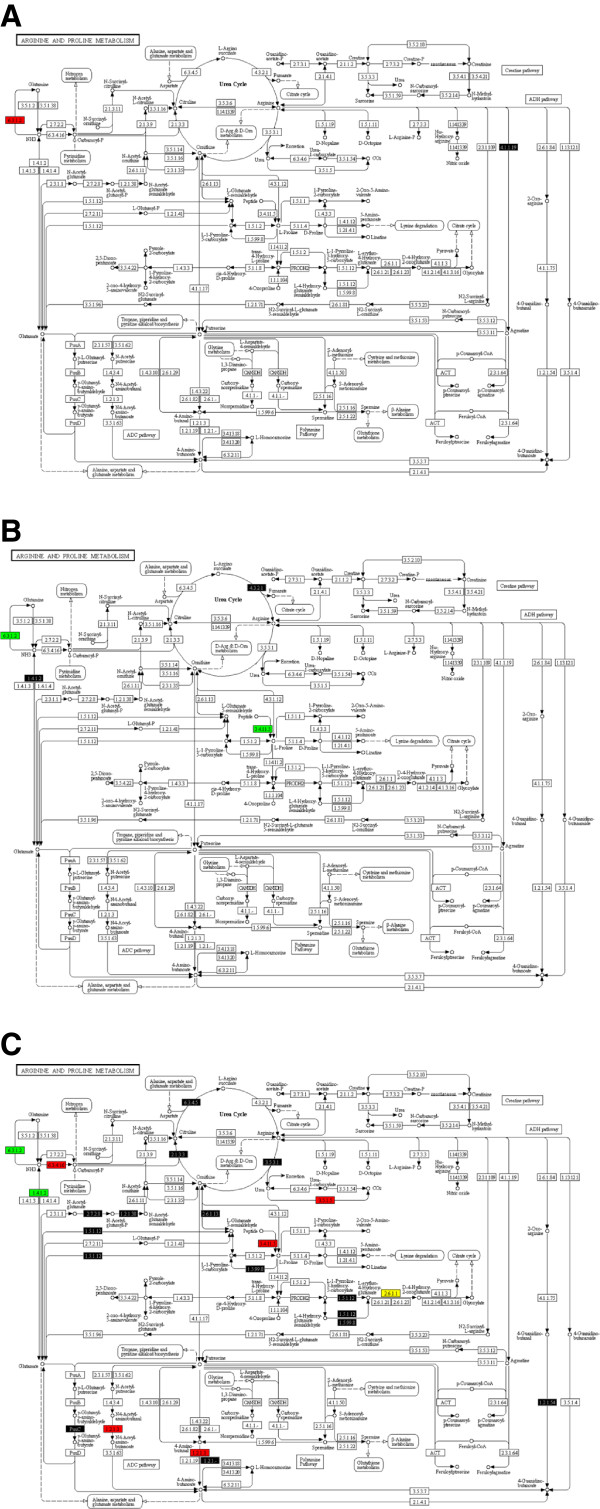
**Representation of arginine and proline metabolism in which regulation status of transcripts encoding enzymes identified at (A) 7 hai, compared to 15 sai, (B) 48 hai, compared to 7 hai, and (C) 10 dai, compared to 48 hai, is annotated.** Color coding as in Figure 1.

Energy, nucleotide metabolism, and protein synthesis are major priorities for the fungus during infection and development. At all-time points, energy production is a necessity for *P. pachyrhizi,* as indicated by expression of many transcripts encoding enzymes involved in energy production such as the expression of genes encoding enzymes of the oxidative phosphorylation pathway, glycolysis, glyoxylate and dicarboxylate metabolism, the pentose phosphate pathway, and pyruvate metabolism. Figure 1 illustrates expression of genes encoding enzymes involved in gluconeogenesis. Fructose-1, 6-bisphosphatase I (E.C. 3.1.3.11) is responsible of the conversion of the beta-D-fructose 1,6-bisphosphate in beta-D-fructose 6-phosphate (Figure 1A). At 15 sai, the transcript level of this gene was high. However, its expression decreased at 7 hai as well as at 48 hai and 10 dai while other enzymes were up-regulated, including those responsible for NADH and ATP synthesis and many more important precursor metabolites throughout the pathway, such as glyceraldehyde-3-phosphate dehydrogenase (E.C. 1.2.1.12) and phosphoglycerate kinase (E.C. 2.7.2.3; Figure 1C). Figure 2 illustrates expression of genes encoding enzymes involved in the oxidative phosphorylation pathway important for energy production. At 15 sai, our transcript abundance data suggest that the major source of energy was coming from reduction of NADH through via complex V of the oxidative phosphorylation pathway, as indicated by high abundance of transcripts encoding ATP synthase (E.C. 3.6.3.14), involved in ATP production through ATP synthase (Figure 2A). However at 7 hai, energy is produced by complex I and IV according to the increased expression level of different NADH dehydrogenases (E.C. 1.6.5.3) and cytochrome -c oxidase (E.C. 1.9.3.1.) (Figure 2A). In comparison, at 48 hai protons were principally produced through complex IV (Figure 2B), according to the abundance of transcripts encoding cytochrome-c oxidase (E.C. 1.9.3.1). Later still, at 10 dai, protons were produced through complexes II, IV and V of the oxidative phosphorylation pathway, according to the abundance of transcripts encoding succinate dehydrogenase (E.C. 1.3.99.1 and 1.3.5.1), cytochrome-c oxidase (E.C. 1.9.3.1), F-type H + −transporting ATPase (E.C. 3.6.3.14), and H + −transporting ATPase (E.C. 3.6.3.6) (Figure 2C). Figure 3 depicts the expression of genes encoding enzymes involved in arginine and proline metabolism. The transcripts encoding enzymes important to this pathway, for example glutamine synthetase (E.C. 6.3.1.2), were down-regulated at 7 hai (Figure 3A) and were up-regulated later at 48 hai and 10 dai (Figure 3B-C). Relative expression levels of genes encoding enzymes involved in these three metabolic pathways (Figures 1, 2 and 3) are color coded as explained in the figure legend.

### Genes encoding enzymes of energy production are abundant in urediniospores

Of the 6,531 contigs identified at 15 sai, 1,054 contigs displayed similarities to DNA sequences in different *P. pachyrhizi* EST libraries. From these, only about 30% shared significant similarity to known proteins.

**Figure 4:**
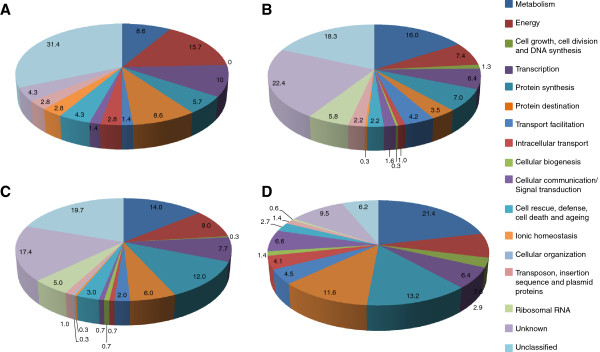
**Functional categorization of potential new *Phakopsora pachyrhizi *transcripts identified at (A) 15 sai, (B) 7 hai, (C) 48 hai, and (D) 10 dai following protein and conserved domain similarity searches against various databases.** The percentage of potential new transcripts found in each category is represented.

Of the remaining 5,477 contigs without similarity to publicly available *P. pachyrhizi* ESTs, our blastx analysis found that only 15% displayed significant similarities to genes encoding proteins listed in the various databases. Figure 4A illustrates the percentage of all identified transcripts sharing similarity to genes encoding fungal proteins belonging to various functional categories.

Even though we collected soybean leaves as early as 15 sai, the urediniospores were not in a quiescent state since they were manipulated during inoculum preparation and plant inoculation. Many fungal transcripts were present. Indeed, many transcripts sharing similarity to genes encoding enzymes involved in energy production were identified, including several encoding electron transport proteins such as the ATP synthase beta subunit (E.C. 3.6.3.14), ferredoxin, NADH dehydrogenase (E.C. 1.6.5.3) subunit 4 and subunit f, cytochrome monohaem, electron carrier oxidoreductase, and fructose-1,6-bisphosphatase (E.C. 3.1.3.11). Transcriptome analysis followed by proteome analysis on dormant *Trychophyton rubrum* conidia indicated that many genes and proteins belonging to glycolysis, the pyruvate dehydrogenase complex, and the oxidative phosphorylation machinery were transcribed [30,31]. These results indicate that transcription and translation of genes involved in energy production and carbohydrate metabolism also occurs earlier than 15 sai. In addition, early studies demonstrated that spore germination was dependent on the function of the standard, cytochrome-mediated electron transport system in *Botryodiplodia theobromae*[32] and *Neurospora crassa*[33], wherein all of the enzyme components in this standard pathway, such as cytochrome-c oxidase and F1F0-type ATP synthase, appeared to be assembled and preserved. These results are consistent with what we observed at 15 sai where transcripts encoding for these enzymes were abundant.

On the other hand, we also identified transcripts at 15 sai that shared similarity to genes encoding proteins involved in transcription, specifically RNA polymerases. Active RNA polymerases have been found previously in ungerminated *Rhizopus stolonifer* spores [34] as well as some other fungi [35] meaning that their presence at 15 sai is not surprising.

**Figure 5:**
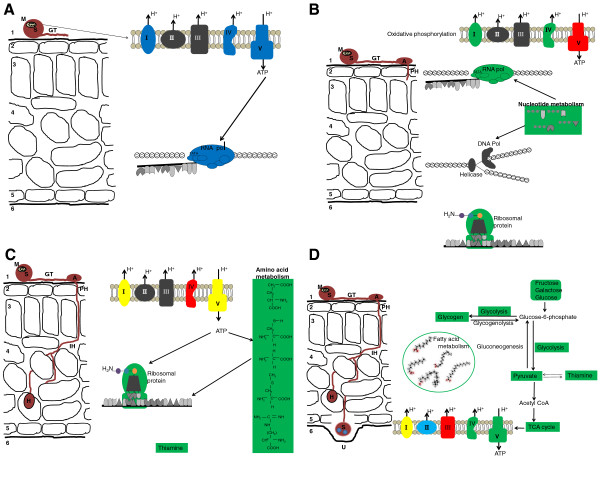
**Schematic representation of events occurring in the pathogen during the infection process; A) 15 sai; B) 7 hai, C) 48 hai, and D) 10 dai of a susceptible soybean leaf with *Phakopsora pachyrhizi*.** Drawing in black represents a cross-section of a soybean leaf where zone 1 represents the upper cuticle, zone 2 the upper epidermis cell layer, zone 3 the palisade mesophyll cell layer, zone 4 the spongy mesophyll cell layer, zone 5 the lower epidermis cell layer, and zone 6 the lower cuticle. Drawing in brown represents *P. pachyrhizi* structures on and inside the soybean leaf showing spore (S), mitochondrion (M), germ tube (GT), appressorium (A), primary hypha (PH), infectious hyphae (IH), haustorium (H), and uredinium (U). Metabolic pathways, proteins, E. C. numbers, products, and substrates colored in blue are activated or expressed; those colored in green are up-regulated compared to the previous time-point; and those colored in red are down-regulated compared to the previous time-point; while boxes colored in yellow represent enzymes encoded by transcripts with varied regulatory status. Metabolic pathways, proteins, E.C. numbers, products, and substrates colored in black were not activated or expressed in the present data set.

Figure 5A is a schematic representation of events occurring into a susceptible soybean leaf about 15 sai with *P. pachyrhizi*. There was abundance of transcripts encoding complexes I, IV, and V involved in oxidative phosphorylation that would be able to produce energy for RNA transcription. Energy may also be produced by other metabolic pathways as indicated by the abundance of transcripts encoding enzymes involved in glycolysis (not shown on the schema). This energy is used by the RNA polymerases to transcribe all genes necessary for the subsequent step of fungal growth.

### Genes encoding enzymes involved in nucleotide metabolism are abundant during urediniospore germination

Of the 4,627 contigs identified at 7 hai representing abundant transcripts during urediniospore germination, 624 contigs displayed similarities to DNA sequences in *P. pachyrhizi* EST libraries. Of the remaining 4,003 contigs without similarity to publicly available *P. pachyrhizi* ESTs, our blastx analysis found that only 15% displayed significant similarities to genes encoding proteins listed in the various databases. Figure 4B illustrates the percentage of all identified transcripts sharing similarity to genes encoding fungal proteins belonging to various functional categories.

A high number of transcripts at 7 hai shared similarity to genes encoding enzymes involved in nucleotide metabolism, such as genes encoding cytosine deaminase, phosphoribosylaminoimidazole carboxylase (E.C. 4.1.1.21), xanthine dehydrogenase (E.C. 1.17.1.4), phosphoribosylformylglycinamidine cyclo-ligase (E. C. 6.3.3.1), and ribonucleoside triphosphate reductase (E.C. 1.17.4.2). Many of these enzymes are directly involved in deoxyribonucleotide and DNA synthesis, while others are involved in aminoimidazole ribotide (AIR) synthesis, which is a precursor of thiamine. Three major classes of macromolecules synthesized rapidly once fungal germination takes place are DNA, RNA and proteins. Interestingly, a pool of transcripts ready to be translated is not always present in ungerminated fungal spores. Indeed, dormant ascospores and conidia of *Neurospora crassa* lack mRNA [36], as do ungerminated conidia of *Fusarium solani*[37], ungerminated basidiospores of *Schizophyllum commune*[38], ungerminated spores of *Dictylostelium purpureum*[39], and dormant conidia of *Penicillium chrysogenum*[40]. The low number of transcripts identified at 15 sai compared to other time-points in the present data set suggests that there is no latent pool of mRNAs present in *P. pachyrhizi* urediniospores as well. Even though the levels of adenosine triphosphate, guanosine triphosphate, cytidine triphosphate, and uridine triphosphate in *P. pachyrhizi* urediniospores are unknown, in other fungi such as dormant spores of *Dictyostelium discoidenum,* the relative levels of these nucleotides is greater than or equal to their levels observed in germinated spores [41]. All together, this suggests that RNA synthesis can occur rapidly after *P. pachyrhizi* urediniospores are deposited on the soybean leaf and even before that point which is consistent with the high level of RNA polymerase expression 15 sai exposed in the previous section. This also occurs during germination of *Rhizopus stolonifer* sporangiospores, where synthesis of all classes of RNA occurred within a few minutes after the sporangiospores were placed on germination medium [42]. Interestingly, the abundance of transcripts encoding enzymes involved in RNA synthesis decreased at 7 hai whereas there was an increase in transcript abundance sharing similarity to genes encoding enzymes involved in DNA synthesis. Proteins encoded by transcripts abundant at 7 hai involved in purine metabolism are exposed in supplementary data (Additional file 1: Figure S1). Along with DNA synthesis, transcripts encoding proteins involved in protein synthesis were lower in abundance at 7 hai, but were expressed, suggesting that protein synthesis also occurred during germination.

Figure 5B is a schematic representation of events occurring into a susceptible soybean leaf at 7 hai with *P. pachyrhizi*. Expression of *P. pachyrhizi* genes encoding F-type H + −transporting ATPase and H + −transporting ATPase (complex V), involved in electron transport is down-regulated compared to their expression at 15 sai while transcript encoding proteins building complexes I and IV are up-regulated. Other metabolic pathways also involved in energy production are similarly down-regulated (data not shown). However, transcription of RNA and synthesis of DNA and protein are active at 7 hai. Transcripts encoding enzymes involved in nucleotide metabolism are abundant at the time DNA synthesis is required by the fungus.

### Genes encoding enzymes involved in amino acid metabolism and protein synthesis are abundant during haustorium formation

Of the 4,273 contigs identified at 48 hai representing abundant transcripts during haustoria formation, 1,130 contigs displayed similarities to DNA sequences in SR EST libraries. As for the two previous time-points, only about 15% of the remaining sequences displayed significant similarities to genes encoding proteins listed in various databases. Figure 4C illustrates the percentage of all transcripts sharing similarity to genes encoding fungal proteins belonging to various functional categories.

Haustoria are important structures for nutrient absorption for many fungi. Evidence of the involvement of haustoria in nutrient uptake from the host cells include increased plasma membrane H^+^-ATPase activity in haustorial membranes as compared to that of membranes from other infection structures [43-45]; preferential expression of genes encoding secondary transporters for amino acids in haustoria [24,46]; and preferential localization of hexose transporter at the tip of differentiating haustoria [47]. However, transcripts sharing similarity to genes encoding membrane transporters and plasma membrane ATPases were not present in our data at 48 hai, which represents the haustoria-formation stage. However, more recent evidences showed that haustoria function not only in nutrient absorption, but also in the suppression of host defense responses, the redirection or reprogramming of the host’s metabolic flow, and in biosynthesis of organic compounds [48].

There is no doubt that fungal pathogens can directly or indirectly reprogram host metabolism, but there is still no proof that this is linked to the function of haustoria. Our data set at 48 hai identified many transcripts sharing similarity to genes encoding proteins involved in amino acid metabolism and protein synthesis, including 5′-methylthioadenosine nucleosidase (E.C. 3.2.2.16), glutamine synthetase, gamma-glutamyl phosphate reductase, cysteine synthase (E.C. 2.5.1.47), methylenetetrahydrofolate reductase and ribosomal proteins. This suggests that protein synthesis occurs during this fungal growth stage in addition to nutrient transport. Proteins encoded by transcripts abundant at 7 hai involved in cysteine and methionine metabolism are exposed in supplementary data (Additional file 2: Figure S2).

Our data from the haustorial stage (48 hai) and that from *P. triticina* haustoria [18] show a limited number of transcripts common to both studies. Of 416 ESTs sharing similarity to genes encoding known proteins identified by Xu et al. [18], only 40% shared similarity to genes encoding fungal proteins and from these, about fifteen transcripts in the present data set at 48 hai shared similarity to genes encoding the same fungal proteins. Eleven additional transcripts shared similarity to genes that were also identified by Yin et al. [26] from a cDNA library from *P. striiformis* f. sp. *tritici* haustoria. They identified approximately 170 ESTs sharing similarity to genes encoding known fungal proteins. Our dataset at 48 hai did not share a lot of commonality with that of Xu et al. [18] and Yin et al. [26] examining haustorial libraries from other fungal species, since they were working on haustoria extracted from plant tissue between 4 and 8 dai. We did not isolate haustoria, but harvested a mix of fungal structures including hyphae and maturing haustoria.

Hyphal walls are mostly made of polysaccharides (60-90%) and some proteins and lipids [49,50]. In contrast, Chong et al. [51] reported that rust haustorial cell walls were made principally of proteins in complexes with polysaccharides and lipids. The high abundance of transcripts sharing similarity to genes encoding enzymes involved in amino acid, lipid, and carbohydrate metabolisms at 48 hai may be related to the synthesis and expansion of hyphal and haustorial walls and membranes.

Not very much is known about the spectrum of biosynthetic reactions occurring in haustoria. However, Hahn and Mendgen [24] found that two of the most abundant genes isolated from their haustorial cDNA library encoded enzymes directly involved in thiamine metabolism. The expression pattern of these genes showed no significant accumulation of transcripts until 18 hours after germination (hag). The transcript abundance increased at 18 and 24 hag and finally accumulated to very high concentrations in haustoria and rust-infected leaves. Thus, *Uromyces fabae* performs thiamine biosynthesis very actively during parasitic growth, presumably because this vitamin is growth limiting and not available from the host plant [24]. In our data set, transcripts sharing similarity to genes encoding a nucleoside triphosphatase (E.C. 3.6.1.15) and a hydrolase from the gdsl-motif lipase hydrolase family (E.C. 3.1.3.-) involved in thiamine metabolism were found at all time-points (Additional file 3: Figure S3). However, their estimated expression was moderate at 15 sai, declined at 7 hai, then increased at 48 hai and increased further by 10 dai. Their estimated expression at 48 hai was not as high as their estimated expression at 15 sai, and at 10 dai their expression was much higher than 15 sai. Thiamine diphosphate coming from pyruvate metabolism is converted to thiamine monophosphate by a nucleoside triphosphatase in presence of water, while the hydrolase from the gdsl-motif lipase hydrolase family converted the thiamine monophosphate into thiamine. There may be another pathway of thiamine production used. Transcripts sharing similarity to genes encoding phosphoribosylformylglycinamidine cyclo-ligase and phosphoribosylaminoimidazole carboxylase/phosphoribosylaminoimidazole-succinocarboxamide synthase were present. Both enzymes are able to convert their respective substrate into aminoimidazole ribotide (AIR) during thiamine metabolism. However, these transcripts were identified only at 7 hai and 10 dai. In addition, no transcript sharing similarity to genes encoding the enzymes responsible for the conversion of AIR into thiamine monophosphate were identified at any time-point. Even though transcripts sharing similarity to genes encoding enzymes involved in pyruvate metabolism were identified, transcripts sharing similarity to genes encoding the pyruvate dehydrogenase E1 component directly responsible for the thiamine diphosphate production was not identified. Unfortunately, there is no known physiological role associated with thiamine monophosphate, but it seems to be important early in *P. pachyrhizi* urediniospore germination and during haustoria maturation through to the end of the infection process.

Figure 5C is a schematic representation of events occurring into a susceptible soybean leaf at 48 hai with *P. pachyrhizi*. There is high abundance of transcripts encoding complexes I and V involved in oxidative phosphorylation that are important for producing energy needed for protein synthesis. Also at 48 hai, transcripts encoding enzymes involved in thiamine metabolism are up-regulated.

### Genes encoding enzymes involved in carbohydrate and fatty acid metabolism are abundant during sporulation

Of the 12,163 contigs identified at 10 dai representing transcripts abundant during urediniospore production, 5,865 contigs displayed similarities to DNA sequences in SR EST libraries. Of the remaining contigs, a blastx analysis found that approximately 25% displayed significant similarities to genes encoding proteins listed in various databases. Figure 4D illustrates the percentage of all transcripts sharing similarity to genes encoding fungal proteins belonging to various functional categories.

At the late stage of infection (10 dai), a high abundance of transcripts sharing similarity to genes encoding enzymes involved in carbohydrate and lipid metabolism was identified. These transcripts included genes encoding almost all enzymes involved in glycolysis and the citrate cycle except ADP-6-phosphofructokinase (E.C. 2.7.1.146). Additional file 4: Figure S4 shows the glycolysis pathway, which includes some enzymes encoded by transcripts that had not been previously identified as abundant during the biotrophic interaction between soybean and *P. pachyrhizi.* Cinq genes encoding enzymes involved in glycolysis are reported here for the first time as being expressed during soybean-*P. pachyrhizi* interaction. They are genes encoding glucose-6-phosphate isomerase, triosephosphate isomerase, phosphoglycerate kinase, pyruvate dehydrogenase E1 component and, hexokinase. Some of these genes that encode a hexokinase were found abundant only at this specific time-point.

Fungal spore formation requires energy that can be produced by metabolism of carbohydrates. Lipids as well as carbohydrates are essential for fungal spore production since they are part of the spore membranes and they are also stored in fungal spores to serve as an energy reserve and a reserve of structural material utilized by the fungus during subsequent germination. Fourteen transcripts involved in lipid metabolism were abundant at 10 dai while not present in earlier time-points. A more detailed description of transcript abundance inside the fungus at 10 dai has previously been described by Tremblay et al. [52].

Figure 5D is a schematic representation of events occurring in a susceptible soybean leaf at 10 dai with *P. pachyrhizi*. There is high abundance of transcripts encoding complexes I, II, IV, and V involved in oxidative phosphorylation to produce energy. Carbohydrate, lipid, and thiamine metabolisms are all activated as indicated by the large number of transcripts encoding enzymes involved in these metabolic pathways at this late time-point.

Combining all time points, there were 3,331 contigs representing *P. pachyrhizi* genes that share similarity to genes encoding known proteins that have not been reported previously in a *P. pachyrhizi*.

### Differential gene expression was confirmed by qPCR

qPCR was conducted using seven representative genes showing a relatively wide range of expression in the present deep mRNA-Seq contig list (Table 3). The expression level of *P. pachyrhizi* alpha-tubulin gene was monitored to confirm that the fungus was developing outside and inside the plant. Primers were designed to amplify transcripts sharing similarity to the gene encoding NADH dehydrogenase subunit f sequenced at 15 sai and 7 hai. The expression level of this gene estimated by deep sequencing showed that it was more highly abundant at 15 sai than at 7 hai. qPCR results were in part consistent with these results, showing that its expression was higher at 15 sai than at 7 hai. However, a PCR product representing transcripts of NADH dehydrogenase subunit f was also detected at 48 hai and 10 dai, at which time-point no transcript encoding the NADH dehydrogenase subunit f was found in our deep-sequencing data. Transcripts encoding a maturase-related protein were found only at 48 hai in our deep-sequencing data. However, qPCR analysis detected transcripts at all time-points and at a higher expression level at 15 sai, 7 hai and 10 dai compared to 48 hai. These two examples illustrate differences that can be found between Illumina RNA-Seq and qPCR. Differences in the detection of transcripts depending upon methodology suggests that not all transcripts are treated equally during sample preparation, conversion into cDNA, amplification, and other steps. In addition, systematic biases have been associated to deep sequencing technology based on variation in the ability to map sequences to the reference genome, because of over-representation of GC-rich sequences compared to AT-rich sequences, and other reasons as suggested by Cheung et al. [53]. The qPCR expression values of all other genes tested were high and correlated well with, mRNA-Seq data. However, qPCR detected the expression of genes at time-points where mRNA-Seq did not, but qPCR expression values were always lower than the expression of these genes at the time-points where mRNA-Seq confirmed gene expression.

**Table 3:** Confirmation of gene expression base on mRNA-Seq assay using qPCR

		**Time-points**			
**Gene description**	**Technique**	**15 sai**	**7 hai**	**48 hai**	**10 dai**
Alpha-tubulin	qPCR^a^	11	30	34	1,848
	mRNA-Seq^b^	NA^c^	NA	NA	1.78
NADH dehydrogenase subunit f	qPCR	293,119	214,031	225,034	548,514
	mRNA-Seq	1.25	0.15	NA	NA
Ribulose-1,5-bisphosphate carboxylase oxygenase large subunit	qPCR	813,754	730,477	708,526	1,794,680
	mRNA-Seq	0.21	NA	NA	1.2
Pectin methylesterase	qPCR	6,726	8,281	5,649	6,488
	mRNA-Seq	NA	0.084	NA	NA
Maturase-related	qPCR	20,598	28,055	15,242	33,772
	mRNA-Seq	NA	NA	0.068	NA
Serine palmitoytransferase	qPCR	0	3	6	609
	mRNA-Seq	NA	NA	NA	0.034
60S ribosomal protein L18	qPCR	0	0	11	1,238
	mRNA-Seq	NA	NA	NA	2.87

^a^Values used to describe qPCR results are absolute number of specific transcript normalized against the absolute number of α-tubulin transcript in the initial sample.

^b^Values used to describe mRNA-Seq results are normalized coverage explain in the Methods section.

^c^Not applicable since the transcript was not sequenced.

## Conclusions

This study identified many new *P. pachyrhizi* abundant transcripts at various stages of fungal development. Even though no information is available describing the function of many of these genes, identification of transcripts specific to different fungal growth stages and their similarity to genes of known function gives us a better understanding of molecular processes occurring during the interaction between *P. pachyrhizi* and soybean plants. In summary our study showed that transcripts encoding proteins sharing similarity to known proteins identified in different databases were mostly (68.4%) down-regulated at 7 hai compared to 15 sai while there was an impressive up-regulation at 48 hai (69.7%) and 10 dai (91.4%). More specifically, energy production is highly active in urediniospores reflected by the abundance of transcripts encoding complexes I, IV, and V involved in oxidative phosphorylation and transcripts encoding proteins involved in glycolysis such as phosphoglucomutase, fructose-1,6-bisphosphatase and triosephosphate isomerase. The energy produced by these metabolic pathways can be used by RNA polymerases, which were also highly abundant, to transcribe genes necessary for the subsequent step of fungal growth. During urediniospores germination, transcripts of genes encoding enzymes involved in nucleotide metabolism was highly abundant along with genes encoding proteins involved in protein synthesis while genes encoding enzymes involved in energy production tended to be down-regulated. At 48 hai, there was an abundance of genes encoding enzymes involved in amino acid metabolism and protein synthesis supporting the recent hypothesis that haustoria, produced during that time frame, play roles in addition to nutrient uptake. There was also abundance of transcripts sharing similarity to genes encoding enzymes involved in lipid metabolism and carbohydrate metabolism that may be associated with the synthesis and expansion of hyphal and haustorial walls and membranes. Finally, at the end of the infection process, transcripts of genes encoding enzymes involved in carbohydrate and fatty acid metabolism were the most highly abundant. Some of these genes and their proteins may potentially serve as targets for developing new modes of plant resistance to fungi by impeding fungal development. Transcripts sharing similarity to genes encoding proteins involved in thiamine metabolism are good examples. These transcripts are abundant 15 seconds after the inoculation process. Their expression decreased at 7 hai but increased at 48 hai and 10 dai when *P. pachyrhizi* is producing haustoria and uredinia, respectively. Fungal thiamine production in haustorium-forming fungi has previously been described as important during haustoria development and may be considered as a good target for disrupting fungal growth and decrease *P. pachyrhizi* infectivity on soybean plants.

## Methods

### Pathogen isolation and plant inoculation

The *P. pachyrhizi* isolate MS06-1 was obtained from urediniospores harvested from field-collected kudzu leaves in Jefferson County, Mississippi, in August 2006. Its identity was confirmed by microscopy, enzyme-linked immunosorbent assay (ELISA) and polymerase chain reaction (PCR) as previously described [54]. Urediniospores were increased on a susceptible soybean cultivar, Williams 82 in the Stoneville Research Quarantine Facility in Mississippi. The isolate was purified by picking a single uredinium using a fine needle under an Olympus SZX12 dissecting microscope and reinoculating it on leaves of Williams 82. This inoculation-isolation cycle was repeated four times. Urediniospores from this purified culture were harvested using a Cyclone Surface Sampler (Burkard Manufacturing Co. Ltd, UK) connected to a vacuum pump at 10 to 14 dai and continuously thereafter at weekly intervals.

Inoculum was prepared using freshly collected urediniospores from Williams 82. Spore suspensions were made using sterile distilled water containing 0.01% Tween-20 (vol/vol) and then filtered through a 100-μm cell strainer (BD Biosciences, Bedford, MA) to remove any debris and clumps of urediniospores. Urediniospores were quantified using a hemocytometer and diluted to a final concentration of 1.1 × 10^5^ spores/mL. Three plants per 10 cm-pot were prepared in three replicates (pots). Primary leaves of 3-weeks-old Williams 82 seedlings were inoculated at a rate of one milliliter of spore suspension per plant using a Preval sprayer (Yonkers, NY). The same solution minus spores was used for a mock inoculation on three pots of plants as a control. After inoculation, plants were placed in a dew chamber in the dark at 22°C overnight (approximately 16 h) and then moved to a Conviron growth chamber where temperatures were maintained at 23°C during the day and 20°C at night under a 16-h photoperiod with a light intensity of 280 μEm^-2^s^-1^. Leaves of soybean cultivar Williams 82 were collected at 15 sai, 7 hai, 48 hai and10 dai with *P. pachyrhizi*. Experiments were repeated once.

### RNA extraction and isolation

Trifoliate leaves from each time-point collected from different pots and replications were pooled in a 50 ml conical tube, fixed in Farmer’s solution and stored at 4°C for shipping from Mississippi to Maryland. One hundred milligrams of leaf pieces from each 50 ml conical tube for each time-point were ground in liquid nitrogen, and RNA was extracted using 450 μl of buffer RLC (Qiagen). RNA was isolated from the sample using an RNeasy Plant Mini Kit (Qiagen) according to the manufacturer’s instructions. RNA was treated on the column with 80 ul of DNA™-free DNAse (Ambion) for 15 min at room temperature. Five hundred nanograms of RNA were used to evaluate its quality and integrity on a 2% agarose gel.

### cDNA preparation

The mRNA was purified from ten micrograms of total RNA using oligo(dT) Dynal magnetic beads (Invitrogen). Two rounds of purification were performed and the resulting mRNA was fragmented at 70°C for five min using RNA fragmentation reagents from Ambion. The fragmented mRNA was precipitated for 30 min at −80°C with 1/10th volume of 3 M NaOAc, 40 μg of glycogen and 3 volumes of 100% EtOH. The fragmented mRNA resuspend in eight microliters of water was used as template for cDNA synthesis. One hundred nanograms of random hexamer were used in concert with the first-strand synthesis reagents from the Superscript III first-strand synthesis system (Invitrogen) following the manufacturer’s instructions. Second-strand synthesis was performed using 1× second-strand buffer (Invitrogen), 0.3 mM of dNTP mix, 2 units of RNaseH and 50 units of DNA polymerase I at 16°C for 2 h and 30 min. cDNA was then purified on a Qiaquick PCR purification column (Qiagen) and eluted in 30 μl of EB buffer (Qiagen). Then, the genomic DNA Sample Preparation Kit from Illumina [55] was used to repair the ends of the cDNA, add a single adenine base, and ligate the adaptor to the cDNA molecules. A 200 +/− 25 bp band was excised from a 2% agarose gel after electrophoresis in 1× TAE buffer for one h at 120 V. The cDNA was purified from the gel using a Qiaquick gel extraction kit (Qiagen) and eluted in 30 μl of EB buffer (Qiagen). The resulting cDNA was PCR enriched using Illumina primers using these conditions: 98°C for 30 sec, 15 cycles of 98°C for 10 sec, 65°C for 30 sec, 72°C for 30 sec and a final step at 72°C for five min.

### Sequencing and contig building

One hundred and twenty microliters of each cDNA library, at eight picomolar (pM), was used to generate clusters on four individual flow cell lanes. A fifth control lane was hybridized with a PhiX Illumina-supplied control.

All four libraries were sequenced using a single-end recipe on the Illumina Genome Analyzer IIx (GAIIx). A set of Cluster Intensity Files (CIF) were produced which were subsequently analyzed using the Illumina Offline Base-Caller (OLB) version 1.6. A break-down of total reads per time-point is presented in Table 1.

CASAVA (Consensus Assessment of Sequence and Variation) then mapped qseq data for all lanes against the twenty soybean chromosomes. Reads aligning to the genome were then mapped against homology-based annotations using TASE (Tag counting and Analysis of Solexa Experiments), producing read-frequencies per annotation [56].

All reads across all four time-points not mapping to the *Glycine max* genome were subsequently put through two rounds of read-subtraction so as to remove contaminant reads be-it other plant or human/bacterial contamination. This subtraction ensures resultant reads to have the greatest likelihood of being potentially *P. pachyrhizi*. For the first subtraction, all reads per time-point were mapped against all available plant genomes from Phytozome [57]. Following, the read-set was then mapped against the JCVI Microbial Database [58] and the NCBI human genome (Hg19). The remaining reads were considered as potential *P. pachyrhizi* (Table 1).

Once both plant-specific and contaminant human/bacterial reads were subtracted from our four read-sets, putative de-novo transcripts were assembled using Velvet and Oases [59,60]. A k-mer hash of 27 and minimum contig length of 75 bp was specified per time-point. Assembled contigs ranged in size from 75 bp to 1,991 bp, composed of 2 to 3,911 reads for any given contig.

Based on the number of homologue reads building a contig, transcript abundance can be estimated as read count and should be relatively proportional to transcript abundance [61,62]. However, read count is vulnerable to sequencing bias since the preparation steps in many of today’s sequencing technologies produce cDNA fragments with positional bias [63]. On the other hand, normalizing coverage, which reflects how many k-mers (a window which yields sub-sequences of length K) map to a specific feature given its full-length, provides a useful numeric in understanding depth of sequencing per feature in an unbiased manner. Given the read length L and k-mer integer K, one can derive a normalized coverage for a given contig:

$$C_{\mathrm{norm}}=\left(C_{\mathrm{kmer}}\right)/\left(L–K+1\right)$$

where C_norm_ represents coverage given the contig length and K = 25 (as used throughout this study).

### Similarity searches

The NCBI nucleotide collection (nr/nt) was downloaded from NCBI [64]. Using BLASTN and the local ‘nr/nt’ database, DNA sequences of all generated contigs were iteratively queried using the MATLAB Bioinformatics Toolbox and an expect-value of 1e-20.

For contigs without similarity to DNA sequences in the NCBI database, a second similarity search strategy was adopted to obtain additional information for those contigs where open reading frames (ORFs) were found. Using the longest ORF for each contig, similarity searches were conducted using translated peptide sequences of contigs against the NCBI non-redundant proteins database, the NCBI conserved domains database (CDD), and the Consortium for the Functional Genomics of Microbial Eukaryotes (COGEME) database (which is specific for phytopathogenic fungi and oomycetes) at an expected-value ≤ 1e-05.

### Confirmation of transcript abundance by quantitative RT-PCR

Template for quantitative RT-PCR (qPCR) was synthesized from RNA (one μg) isolated from a pool of 100 mg of infected leaf tissue by converting it to cDNA using SuperScript First-Strand Synthesis System for RT-PCR (Invitrogen) with an oligo(dT) as a primer according to the manufacturer’s instructions. Ten ng of cDNA were used in a 25 μl reaction containing 1× of Brilliant SYBR Green qPCR master mix (Stratagene) and 0.15 μM of primers. Primers amplifying the *P. pachyrhizi* α-tubulin [65] as well as seven additional *P. pachyrhizi* transcripts abundant at various fungal growth stages were used to support deep sequencing results (Table 4). The cycling conditions consisted of the following steps: an initial 15 min denaturing step at 95°C; 50 cycles at 95°C for 10 sec, and 65°C for 2 min. A dissociation curve analysis was performed for detection of non-specific products if any.

**Table 4:** PCR primer pairs

**Name**	**Encoded protein**	**Sequence**	**Size (bp)**
NADHf-7	NADH dehydrogenase subunit f	Forward: TCCCAGACACGATTAGTTACAAATGCT	107
		Reverse: TGGGAATTGGTTGGAATGTG	
60SRPL18-10	60S ribosomal protein L18	Forward: GCCCTCAGACACCCTACCG	168
		Reverse: ACCTCGCGATGCTCTTCTTC	
RBCOlu-0	ribulose-1,5-bisphosphate carboxylase / oxygenase large subunit	Forward: CGGTATTTATTTCACTCAGGATTGGGT	104
		Reverse: CAAAGATCTCGGTCAGAGCAGGC	
PME-7	Pectin methylesterase	Forward: CTCGTGGATGGTTGGAGTGGA	105
		Reverse: CATTGAACCCGTTGGCCCAC	
Mat-48	Maturase-related protein	Forward: ACCAATTTACGATGTCTCCGTCGC	135
		Reverse:CTATACAGATAGAGGCGCCTATCAAAAAG	
SPT-10	Serine palmitoyltransferase	Forward:GAGGAGTATGCGATTACTATGGAGTTG	68
		Reverse: CTTTGTCAGAGTTCCCATCAAGAT	
αTUB	Alpha-tubulin	Forward: CCAAGGCTTCTTCGTGTTTCA	65
		Reverse: CAAGAGAAGAGCGCCAAACC	

All primer sets were designed from regions that flanked an intron to make sure that expected size product was amplified from cDNA and not from genomic DNA. An additional control tube containing no template was included for each specific reaction using different primer sets.

qPCRs using all primer sets were performed as three technical replicates. Relative levels of gene expression were determined using the Stratagene Mx3000P Real-Time PCR system (Stratagene, La Jolla, CA) as described by the manufacturer. DNA accumulation during the reaction was measured with SYBR Green. The Ct (cycle at which there is the first clearly detectable increase in fluorescence) values were calculated using software supplied with the Stratagene Mx3000P Real-Time PCR system. The SYBR green dissociation curve of the amplified products demonstrated the production of only one product per reaction. Data analysis was performed according to the sigmoidal model [66] to get absolute quantification as described in Tremblay et al. [28].

### Availability of supporting data

The data sets supporting the results of this article are available in the Sequence Read Archive repository at the National Center for Biotechnology Information, SRR445529 (http://www.ncbi.nlm.nih.gov/sra/?term=SRR445529), SRR610280 (http://www.ncbi.nlm.nih.gov/sra/?term=SRR610280), SRR610284 (http://www.ncbi.nlm.nih.gov/sra/?term=SRR6102804), SRR445528 (http://www.ncbi.nlm.nih.gov/sra/?term=SRR445528).

## Competing interests

The authors declare that they have no competing interests.

## Authors’ contributions

AT, BFM conceived the experiments; SL provided the infected materials; AT performed the experiments; AT, PH analyzed the data; AT wrote the manuscript; PH, SL, NWA, BFM edited the manuscript. All authors read and approved the final manuscript.

## Supplementary Material

Additional file 1: Figure S1 Representation of purine metabolism showing enzymes encoded by transcripts identified at 7 hai. Boxes colored in orange represent enzymes encoded by only newly identified transcripts at this specific time-point. Click here for file

Additional file 2: Figure S2 Representation of cysteine and methionine metabolism showing enzymes encoded by transcripts identified at 48 hai. Boxes colored in orange represent enzymes encoded by only newly identified transcripts. Click here for file

Additional file 3: Figure S3 Representation of thiamine metabolism over the time-course of infection: A) 15 sai, B) 7 hai, C) 48 hai, and D) 10 dai. E.C. numbers colored in blue are activated or expressed; those colored in green are up-regulated compared to the previous time-point; and those colored in red are down-regulated compared to the previous time-point. Metabolic pathways, proteins, E.C. numbers, products, and substrates colored in black were not activated or expressed in the present data set. Click here for file

Additional file 4: Figure S4 Representation of glycolysis showing enzymes encoded by transcripts identified at 10 dai. Color coding as in Additional file 1: Figure S1, with the addition of boxes colored in pink representing enzymes encoded by both newly identified transcripts specific to 10 dai and transcripts sharing similarity to previously identified *P. pachyrhizi* ESTs and boxes colored in purple represent enzymes encoded only by transcripts sharing similarity to previously identified *P. pachyrhizi* ESTs. Click here for file

## References

[B1] Lynch TN Marois JJ Wright DL Harmon PF Harmon CL Miles MR Hartman GL First report of soybean rust caused by *Phakopsora pachyrhizi* on *Phaseolus* spp. in the United States Plant Dis 2006 90 970 10.1094/PD-90-0970C 30781041

[B2] Ono Y Buritica P Hennen JF Delimitation of *Phakopsora*, *Physopella* and *Cerotelium* and their species on *Leguminosae* Mycol Res 1992 96 825 850 10.1016/S0953-7562(09)81029-0

[B3] Rytter JL Dowler WM Bromfield KR Additional alternative hosts of *Phakopsora pachyrhizi*, causal agent of soybean rust Plant Dis 1984 68 818 819

[B4] Sconyers LE Kemerait RC Brock JH Gitaitis RD Sanders FH Phillips DV Jost PH First report of *Phakopsora pachyrhizi*, the causal agent of Asian Soybean Rust, on Florida beggarweed in the United States Plant Dis 2006 90 972 10.1094/PD-90-0972A 30781045

[B5] Miles MR Levy C Morel W Mueller T Steinlage T van Rij N Frederick RD Hartman GL International fungicide efficacy trials for the management of soybean rust Plant Dis 2007 91 1450 1458 10.1094/PDIS-91-11-1450 30780750

[B6] Yeh CC Yang CY Yield loss cause by soybean rust, Phakopsora pachyrhizi Plant Prot Bull (Taiwan ROC) 1975 17 7 8

[B7] Yorinori JT Pavia WM Frederick RD Costamilan LM Bertagnolli PF Hartman G Godoy C Nunes J Jr Epidemics of soybean rust (*Phakopsora pachyrhizi*) in Brazil and Paraguay from 2001 to 2003 Phytopathology 2003 93 S103 10.1094/PD-89-0675 30795398

[B8] Sinclair JB Hartman GL Hartman GL, Sinclair JB, Rupe JC Soybean rust Compendium of soybean diseases 1999 St. Paul, MN: American Phytopathological Society

[B9] Mississippi Agricultural and Forestry Experimental Station, Mississippi State University Extension Servicehttp://msucares.com/crops/soybeans/rust/index.html

[B10] Calvo ES Kiihl RAS Garcia A Harada A Hiromoto DM Two major recessive soybean genes conferring soybean rust resistance Crop Sci 2008 48 1350 1354 10.2135/cropsci2007.10.0589

[B11] Meyer JDF Silva DCG Yang C Pedley KF Zhang C van de Mortel M Hill JH Shoemaker RC Abdelnoor RV Whitham SA Graham MA Identification and analyses of candidate genes for *Rpp4*-mediated resistance to Asian soybean rust in soybean Plant Physiol 2009 150 295 307 10.1104/pp.108.134551 19251904 PMC2675740

[B12] Posada-Buitrago ML Frederick RD Expressed sequence tag analysis of the soybean rust pathogen *Phakopsora pachyrhizi* Fungal Genet Biol 2005 42 949 962 10.1016/j.fgb.2005.06.004 16291502

[B13] Li S Smith JR Ray JD Frederick RD Identification of a new soybean rust resistance gene in PI 567102B Theor Appl Genet 2012 125 133 142 10.1007/s00122-012-1821-y 22374138

[B14] Hartwig EE Identification of a fourth major gene conferring resistance to soybean rust Crop Sci 1986 26 1135 1136 10.2135/cropsci1986.0011183X002600060010x

[B15] Monteros MJ Ha BK Phillips DV Boerma HR SNP assay to detect the ‘Hyuuga’ red-brown lesion resistance gene for Asian soybean rust Theor Appl Genet 2010 121 1023 1032 10.1007/s00122-010-1368-8 20532750 PMC2938421

[B16] Stone CL McMahon MB Fortis LL Nunez A Smythers GW Luster DG Frederick RD Gene expression and proteomic analysis of the formation of *Phakopsora pachyrhizi* appressoria BMC Genomics 2012 13 269 289 10.1186/1471-2164-13-269 22727213 PMC3431228

[B17] Zhang Y Qu Z Zheng W Liu B Wang X Xue X Xu L Huang L Han Q Zhao J Kang Z Stage-specific gene expression during urediniospore germination in *Puccinia striiformis* f. sp *tritici* BMC Genomics 2008 9 203 10.1186/1471-2164-9-203 18447959 PMC2386484

[B18] Xu J Linning R Fellers J Dickinson M Zhu W Antonov I Joly DL Donaldson ME Eilam T Anikster Y Banks T Munro S Mayo M Wynhoven B Ali J Moore R McCallum B Borodovsky M Saville B Bakkeren G Gene discovery in EST sequences from the wheat leaf rust fungus *Puccinia triticina* sexual spores, asexual spores and haustoria, compared to other rust and corn smut fungi BMC Genomics 2011 12 161 10.1186/1471-2164-12-161 21435244 PMC3074555

[B19] Zahiri AR Babu MR Saville BJ Differential gene expression during teliospore germination in *Ustilago maydis* Mol Genet Genomics 2005 273 394 403 10.1007/s00438-005-1142-9 15887033

[B20] Deng Y Dong H Jin Q Dai C Fang Y Liang S Wang K Shao J Lou Y Shi W Vakalounakis DJ Li D Analysis of expressed sequence Tag data and gene expression profiles involved in conidial germination of *Fusarium oxysporum* Appl Environ Microb 2006 72 1667 1671 10.1128/AEM.72.2.1667-1671.2006 PMC1392917 16461724

[B21] Hahn M Mendgen K Isolation by ConA binding of haustoria from different rust fungi and comparison of their surface qualities Protoplasma 1992 170 95 103 10.1007/BF01378785

[B22] Loehrer M Schaffrath U Tzi-Bun N Asian soybean rust – meet a prominent challenge in soybean cultivation, soybean Soybean - Biochemistry, Chemistry and Physiology 2011 Croatia: InTech

[B23] Catanzariti A-M Dodds PN Lawrence GJ Ayliffe MA Ellis JG Haustorially expressed secreted proteins from flax rust are highly enriched for avirulence elicitors Plant Cell 2006 18 243 256 10.1105/tpc.105.035980 16326930 PMC1323496

[B24] Hahn M Mendgen K Characterization of In Planta–Induced Rust Genes Isolated from a Haustorium-Specific cDNA Library Mol Plant Microbe 1997 10 427 437 10.1094/MPMI.1997.10.4.427 9150592

[B25] Puthoff DP Neelam A Ehrenfried ML Scheffler BE Ballard L Song Q Campbell KB Cooper B Tucker ML Analysis of expressed sequence tags from *Uromyces appendiculatus* hyphae and haustoria and their comparison to sequences from other rust fungi Phytopathology 2008 98 1126 1135 10.1094/PHYTO-98-10-1126 18943459

[B26] Yin C Chen X Wang X Han Q Kang Z Hulbert SH Generation and analysis of expression sequence tags from haustoria of the wheat stripe rust fungus *Puccinia striiformis* f. sp. *Tritici* BMC Genomics 2009 10 626 10.1186/1471-2164-10-626 20028560 PMC2805700

[B27] Marchetti MA Uecker FA Bromfield KR Uredial development of *Phakopsora pachyrhizi* in soybeans Phytopathology 1975 65 822 823 10.1094/Phyto-65-822

[B28] Tremblay A Li S Scheffler BE Matthews BF Laser capture microdissection and expressed sequence tag analysis of uredinia formed by *Phakopsora pachyrhizi*, the causal agent of Asian soybean rust Physiol Mol Plant P 2009 73 163 174

[B29] Hacquard S Delaruelle C Legué V Tisserant E Kohler A Frey P Martin F Duplessis S Laser capture microdissection of uredinia formed by Melampsora larici-populina revealed a transcriptional switch between biotrophy and sporulation Mol Plant Microbe In 2010 23 1275 1286 10.1094/MPMI-05-10-0111 20831407

[B30] Leng W Liu T Li R Yang J Wei C Zhang W Jin Q Proteomic profile of dormant *Trichophyton Rubrum* conidia BMC Genomics 2008 9 303 10.1186/1471-2164-9-303 18578874 PMC2443143

[B31] Liu T Zhang Q Wang L Yu L Leng W Yang J Chen L Peng J Ma L Dong J Xu X Xue Y Zhu Y Zhang W Yang L Li W Sun L Wan Z Ding G Yu F Tu K Qian Z Li R Shen Y Li Y Jin Q The use of global transcriptional analysis to reveal the biological and cellular events involved in distinct development phases of *Trichophyton rubrum* conidial germination BMC Genomics 2007 8 100 10.1186/1471-2164-8-100 17428342 PMC1871584

[B32] Wenzler H Brambl R Mitochondrial biogenesis during fungal spore germination. Catalytic activity, composition, and subunit biosynthesis of oligomycin-sensitive ATPase in Botryodiplodia J Biol Chem 1981 256 7166 7172 6454687

[B33] Stade S Brambl R Mitochondrial biogenesis during fungal spore germination: respiration and cytochrome c oxidase in *Neurospora crassa* J Bacteriol 1981 147 757 767 6268605 10.1128/jb.147.3.757-767.1981 PMC216111

[B34] Gong C-S Van Etten JI Changes in soluble ribonucleic acid polymerases associated with the germination of *Rhizopus stolonifer* spores Biochimica and Biophysica Acta 1972 272 44 52 10.1016/0005-2787(72)90031-7 5043713

[B35] Van Etten JL Dunkle LD Knight RH Weber DJ, Hess WH Nucleic acids and fungal spore germination The fungal spore: form and function 1976 New York: John Wiley & Sons 243 300

[B36] Henney HR Jr Storck R Ribosomes and ribonucleic acids in three morphological states of Neurospora Science 1963 142 1675 1677 10.1126/science.142.3600.1675 14075701

[B37] Rado TA Cochrane VW Ribosomal Competence and Spore Germination in *Fusarium solani* J Bacteriol 1971 106 301 304 5573727 10.1128/jb.106.2.301-304.1971 PMC285096

[B38] Leary JV Morris AJ Ellingboe AE Isolation of functional ribosomes and polysomes from lyophilized fungi Biochimica and Biophysica Acta 1969 182 113 120 10.1016/0005-2787(69)90526-7 5792845

[B39] Feit IN Chu LK Iverson RM Appearance of polyribosomes during germination of spores in cellular slime mold *Dictyostelium purpureum* Exp Cell Res 1971 65 439 444 10.1016/0014-4827(71)90024-3 4928804

[B40] Kornfield JM Structural and physiological aspects of germination of conidia of Penicillium chrysogenum PhD thesis 1961 Madison: University of Wisconsin

[B41] Hamer JE Cotter DA The timing of ribonucleic acid synthesis during the germination of heat-activated *Dictyostelium discoideum* spores Can J Microbiol 1983 29 1390 1398 10.1139/m83-214 6661701

[B42] Roheim JR Knight RH Van Etten JL Synthesis of ribonucleic acids during germination of *Rhizopus stolonifer* sporangiospores Dev Biol 1974 41 137 145 10.1016/0012-1606(74)90289-9 4435301

[B43] Hahn M Deising H Struck C Mendgen K Hartleb H, Heitefuss R, Hoppe H-H Fungal morphogenesis and enzyme secretion during pathogenesis Resistance of crop plants against fungi 1997 Germany: Gustav Fischer Verlag 33 57

[B44] Struck C Hahn M Mendgen K Plasma membrane H + −ATPase activity in spores, germ tubes, and haustoria of the rust fungus *Uromyces viciae-fabae* Fungal Genet Biol 1996 20 30 35 10.1006/fgbi.1996.0006 8812284

[B45] Struck C Siebels C Rommel O Wernitz M Hahn M The plasma membrane H + −ATPase from the biotrophic rust fungus *Uromyces fabae*: molecular characterization of the gene (*PMA1*) and functional expression of the enzyme in yeast Mol Plant Microbe In 1998 11 458 465 10.1094/MPMI.1998.11.6.458 9612944

[B46] Hahn M Neef U Struck C Göttfert M Mendgen K A putative amino acid transporter is specifically expressed in haustoria of the rust fungus *Uromyces fabae* Mol Plant Microbe In 1997 10 438 445 10.1094/MPMI.1997.10.4.438 9150593

[B47] Voegele RT Struck C Hahn M Mendgen K The role of haustoria in sugar supply during infection of broad bean by the rust fungus *Uromyces fabae* Proc Natl Acad Sci USA 2001 98 8133 8138 10.1073/pnas.131186798 11390980 PMC35480

[B48] Voegele RT Mendgen K Rust haustoria: nutrient uptake and beyond New Phytol 2003 159 93 100 10.1046/j.1469-8137.2003.00761.x 33873671

[B49] Bartnicki-Garcia S Chemistry of hyphal walls of *Phytophthora* J Gen Microbiol 1966 42 57 69 10.1099/00221287-42-1-57 5922299

[B50] Chet I Henis Y Chemical composition of hyphal and sclerotial walls of *Sclerotium rolfsii* sacc Can J Microbiol 1967 13 137 141 10.1139/m67-019 6035531

[B51] Chong J Harder DE Rohringer R Ontogeny of mono- and dikaryotic rust haustoria: cytochemical and ultrastructural studies Phytopathology 1981 71 975 983 10.1094/Phyto-71-975

[B52] Tremblay A Hosseini P Li S Alkharouf NW Matthews BF Identification of genes expressed by *Phakopsora pachyrhizi*, the pathogen causing soybean rust, at a late stage of infection of susceptible soybean leaves Plant Pathol 2011 61 773 786

[B53] Cheung M-S Down TA Latorre I Ahringer J Systematic bias in high-throughput sequencing data and its correction by BEADS Nucleic Acids Res 2011 39 e103 10.1093/nar/gkr425 21646344 PMC3159482

[B54] Li S Moore WF Spinks BL Wells BC Sciumbato GL Robinson SJ Libous-Bailey L Occurrence of Asian soybean rust caused by *Phakopsora pachyrhizi* in Mississippi Plant Health Prog 2007 doi:10.1094/PHP-2007-0917-02-BR

[B55] Illuminahttp://www.illumina.com

[B56] Hosseini P Tremblay A Matthews BF Alkharouf NW An efficient annotation and gene-expression derivation tool for Illumina Solexa Datasets BMC Res Notes 2010 3 183 10.1186/1756-0500-3-183 20598141 PMC2908109

[B57] Phytozomehttp://www.phytozome.net/

[B58] Davidsen T Beck E Ganapathy A Montgomery R Zafar N Yang Q Madupu R Goetz P Galinsky K White O Sutton G The comprehensive microbial resource Nucleic Acids Res 2010 38 database issue D340 D345 19892825 10.1093/nar/gkp912 PMC2808947

[B59] Zerbino DR Birney E Velvet: Algorithms for *de novo* short read assembly using de Bruijn graphs Genome Res 2008 18 821 829 10.1101/gr.074492.107 18349386 PMC2336801

[B60] Schulz MH Zerbino D Vingron M Birney E Oases: robust de novo RNA-seq assembly across the dynamic range of expression levels Nucleic Acids Res 2012 28 1086 1092 10.1093/bioinformatics/bts094 PMC3324515 22368243

[B61] Balwierz PJ Carninci P Daub CO Kawai J Hayashizaki Y Van Belle W Beisel C van Nimwegen E Methods for analyzing deep sequencing expression data: constructing the human and mouse promoteome with deepCAGE data Genome Biol 2009 10 R79 10.1186/gb-2009-10-7-r79 19624849 PMC2728533

[B62] Mortazavi A Williams BA McCue K Schaeffer L Wold B Mapping and quantifying mammalian transcriptomes by RNA-Seq Nat Methods 2008 5 621 628 10.1038/nmeth.1226 18516045 PMC13303166

[B63] Roberts A Trapnell C Donaghey J Rinn JL Pachter L Improving RNA-Seq expression estimates by correcting for fragment bias Genome Biol 2011 12 R22 10.1186/gb-2011-12-3-r22 21410973 PMC3129672

[B64] FTP rootftp.ncbi.nih.gov

[B65] van de Mortel M Recknor JC Graham MA Nettleton D Dittman JD Nelson RT Godoy CV Abdelnoor RV Almeida AMR Baum TJ Whitham SA Distinct biphasic mRNA changes in response to Asian soybean rust infection Mol Plant Microbe In 2007 20 887 899 10.1094/MPMI-20-8-0887 17722693

[B66] Rutledge RG Stewart D A kinetic-based sigmoidal model for the polymerase chain reaction and its application to high-capacity absolute quantitative real-time PCR BMC Biotechnol 2008 8 47 10.1186/1472-6750-8-47 18466619 PMC2397388

