# Supplementary figures and images for: Analysis of Phakopsora pachyrhizi transcript abundance in critical pathways at four time-points during infection of a susceptible soybean cultivar using deep sequencing
Source: BMC Genomics. 2013 Sep 11;14:614. doi: 10.1186/1471-2164-14-614 (PMC3847679; doi:10.1186/1471-2164-14-614)

# PURINE METABOLISM

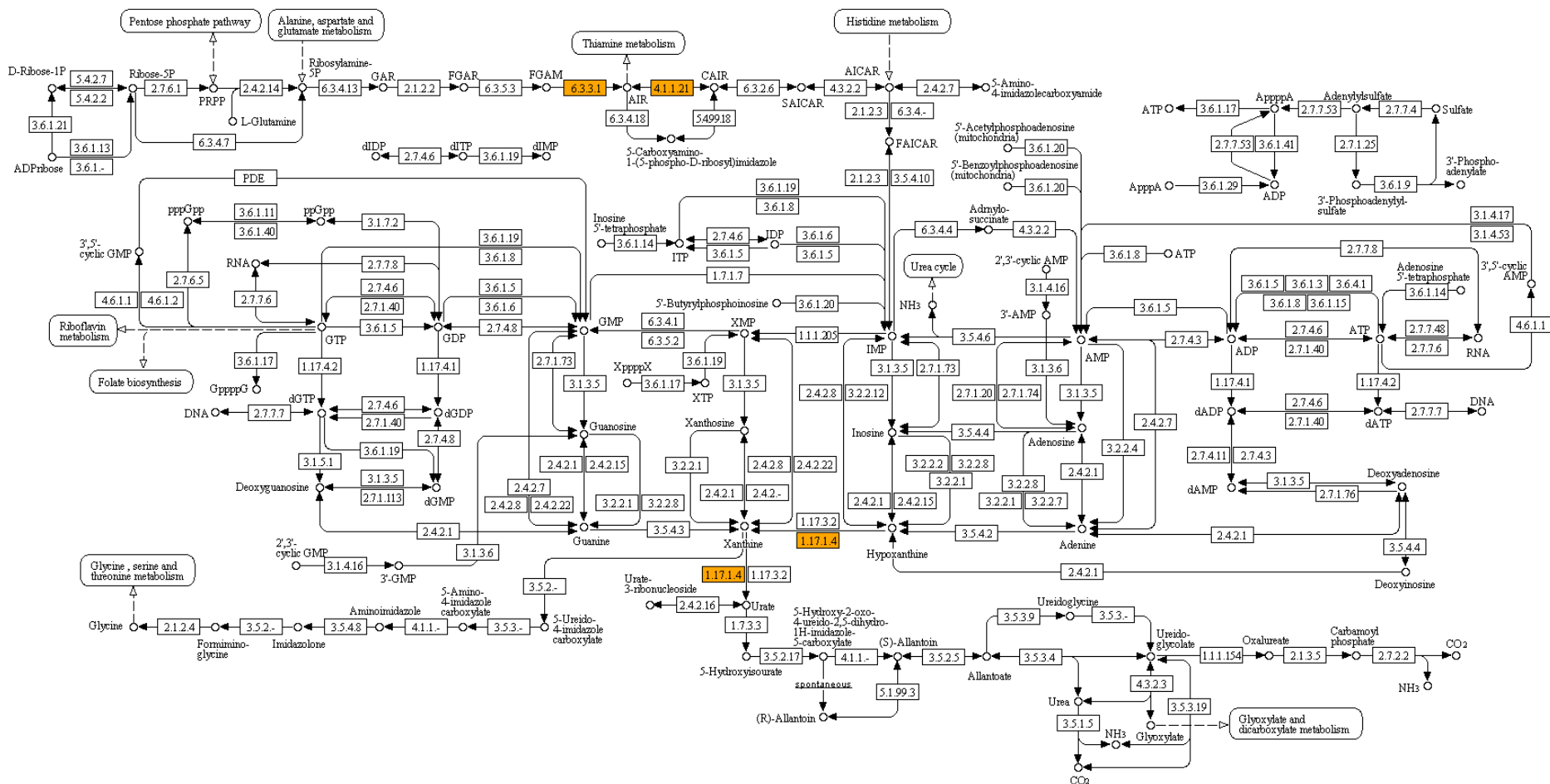

Supplement: Additional file 1: Figure S1 — Representation of purine metabolism showing enzymes encoded by transcripts identified at 7 hai. Boxes colored in orange represent enzymes encoded by only newly identified transcripts at this specific time-point. [file 1471-2164-14-614-S1.pdf]

## CYSTEINE AND METHIONINE METABOLISM

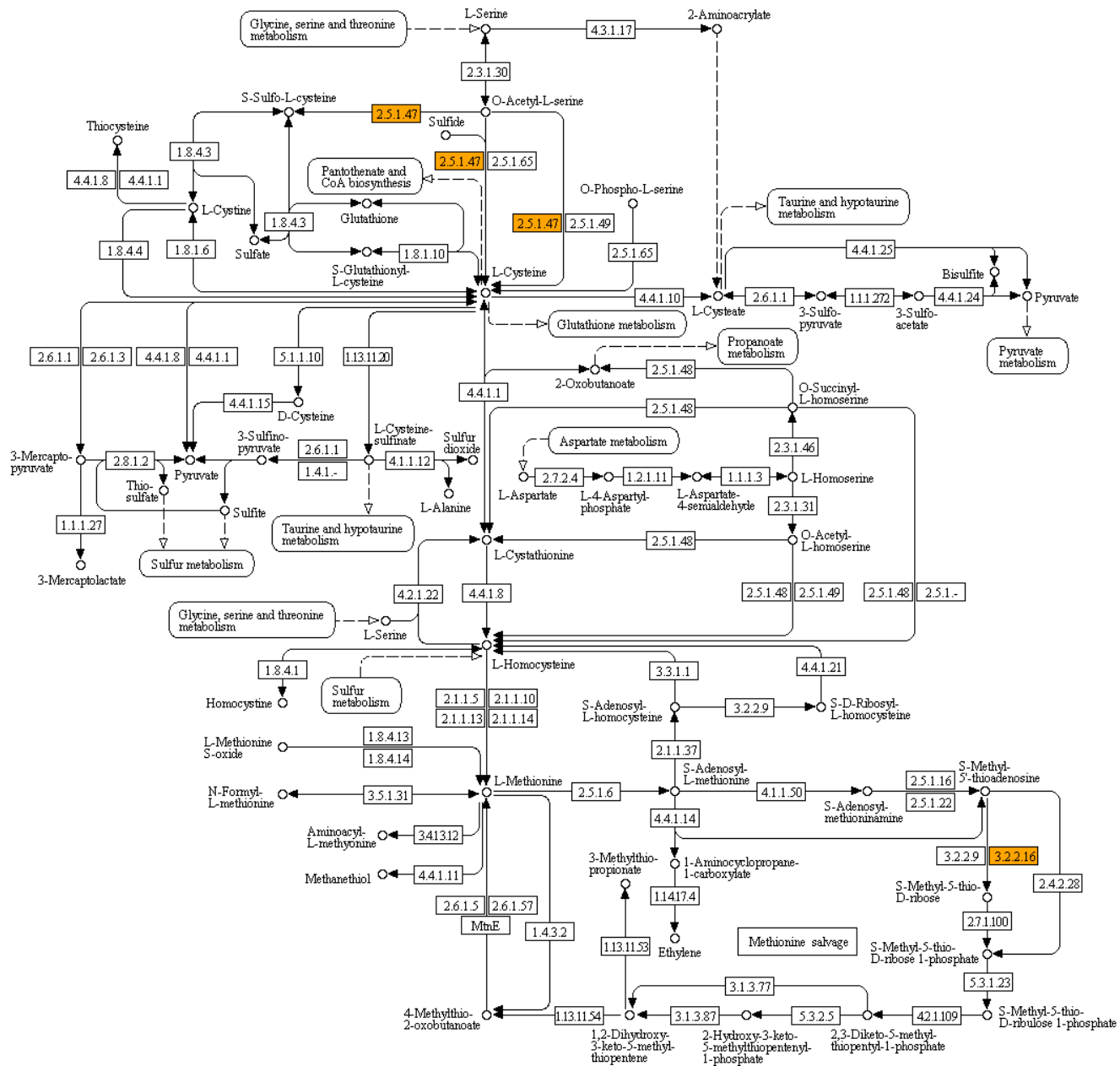

Supplement: Additional file 2: Figure S2 — Representation of cysteine and methionine metabolism showing enzymes encoded by transcripts identified at 48 hai. Boxes colored in orange represent enzymes encoded by only newly identified transcripts. [file 1471-2164-14-614-S2.pdf]

A

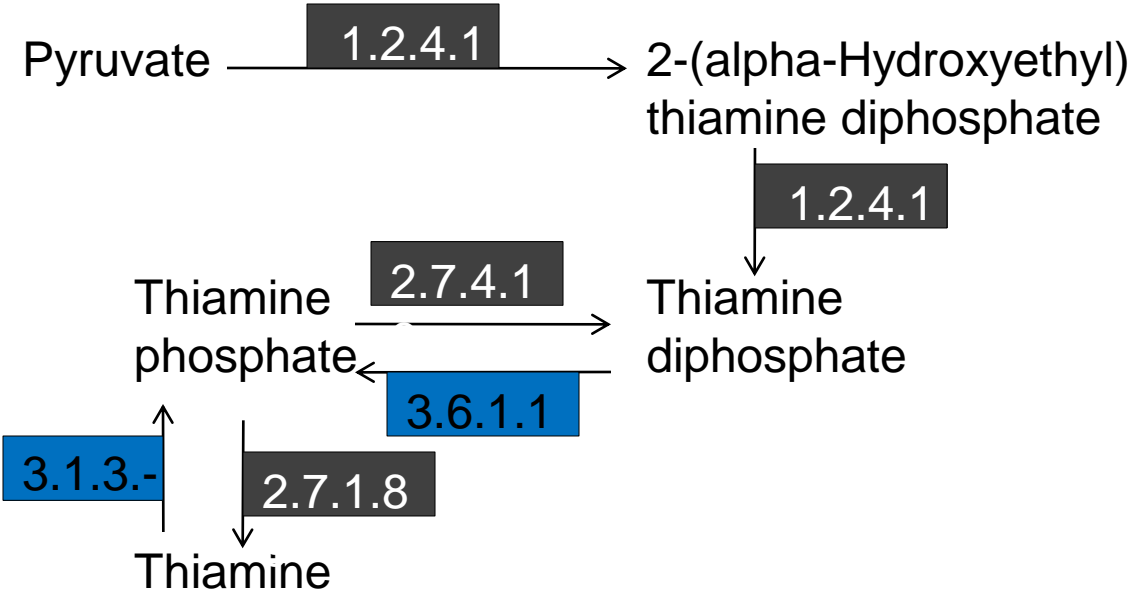

B

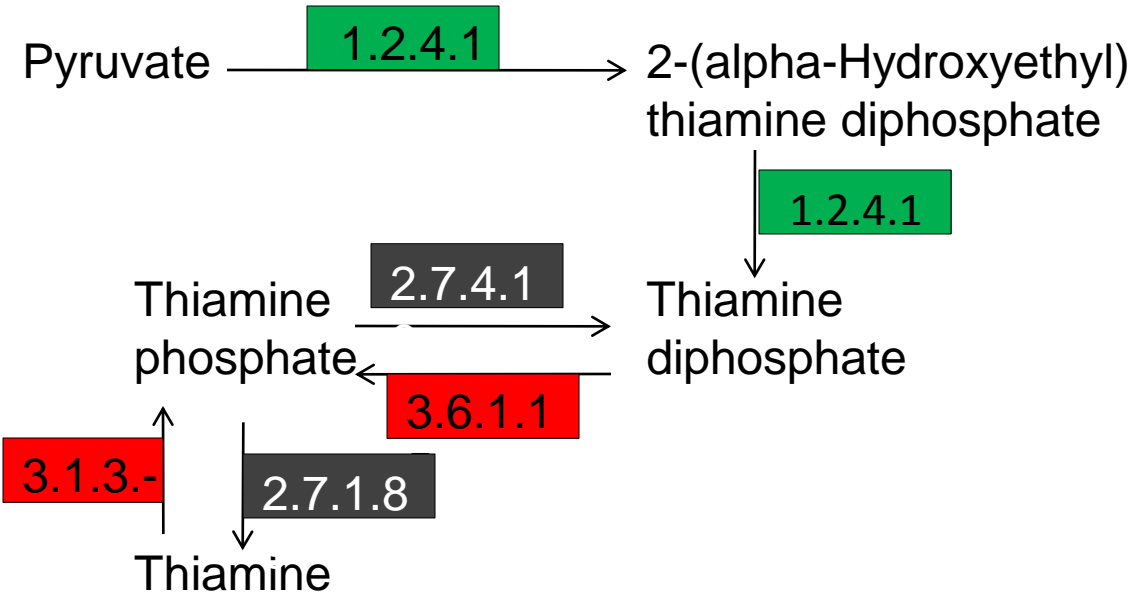

C

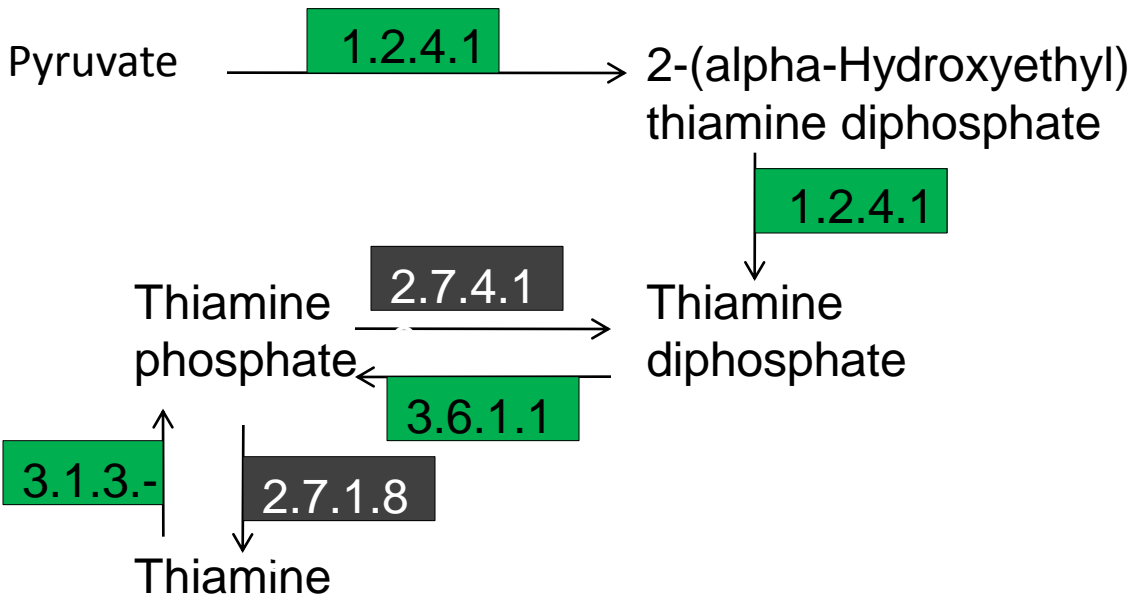

D

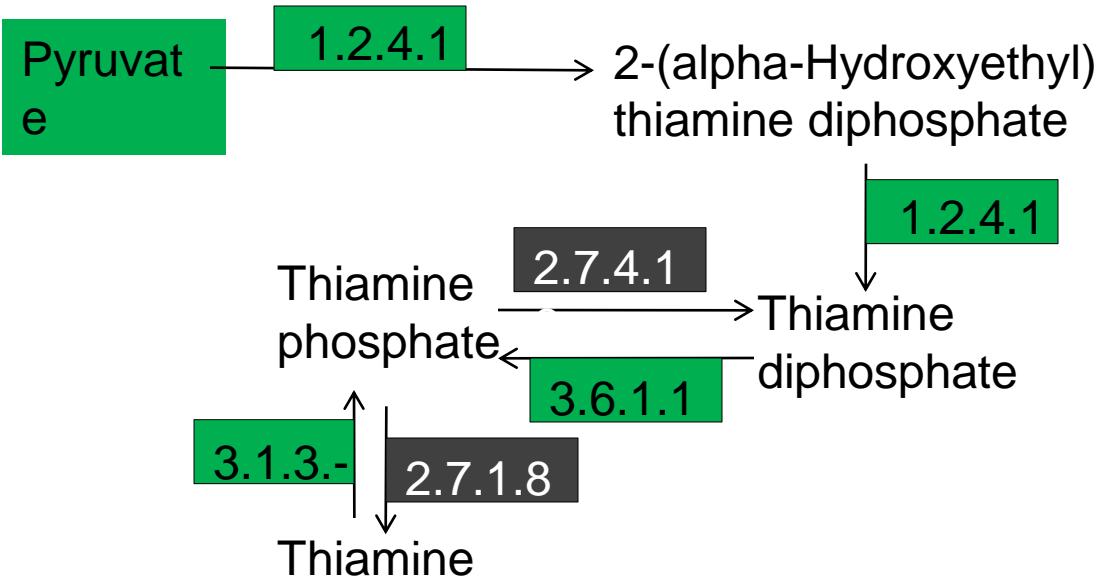

Supplement: Additional file 3: Figure S3 — Representation of thiamine metabolism over the time-course of infection: A) 15 sai, B) 7 hai, C) 48 hai, and D) 10 dai. E.C. numbers colored in blue are activated or expressed; those colored in green are up-regulated compared to the previous time-point; and those colored in red are down-regulated compared to the previous time-point. Metabolic pathways, proteins, E.C. numbers, products, and substrates colored in black were not activated or expressed in the present data set. [file 1471-2164-14-614-S3.pdf]

GLYCOLYSIS / GLUCONEOGENESIS

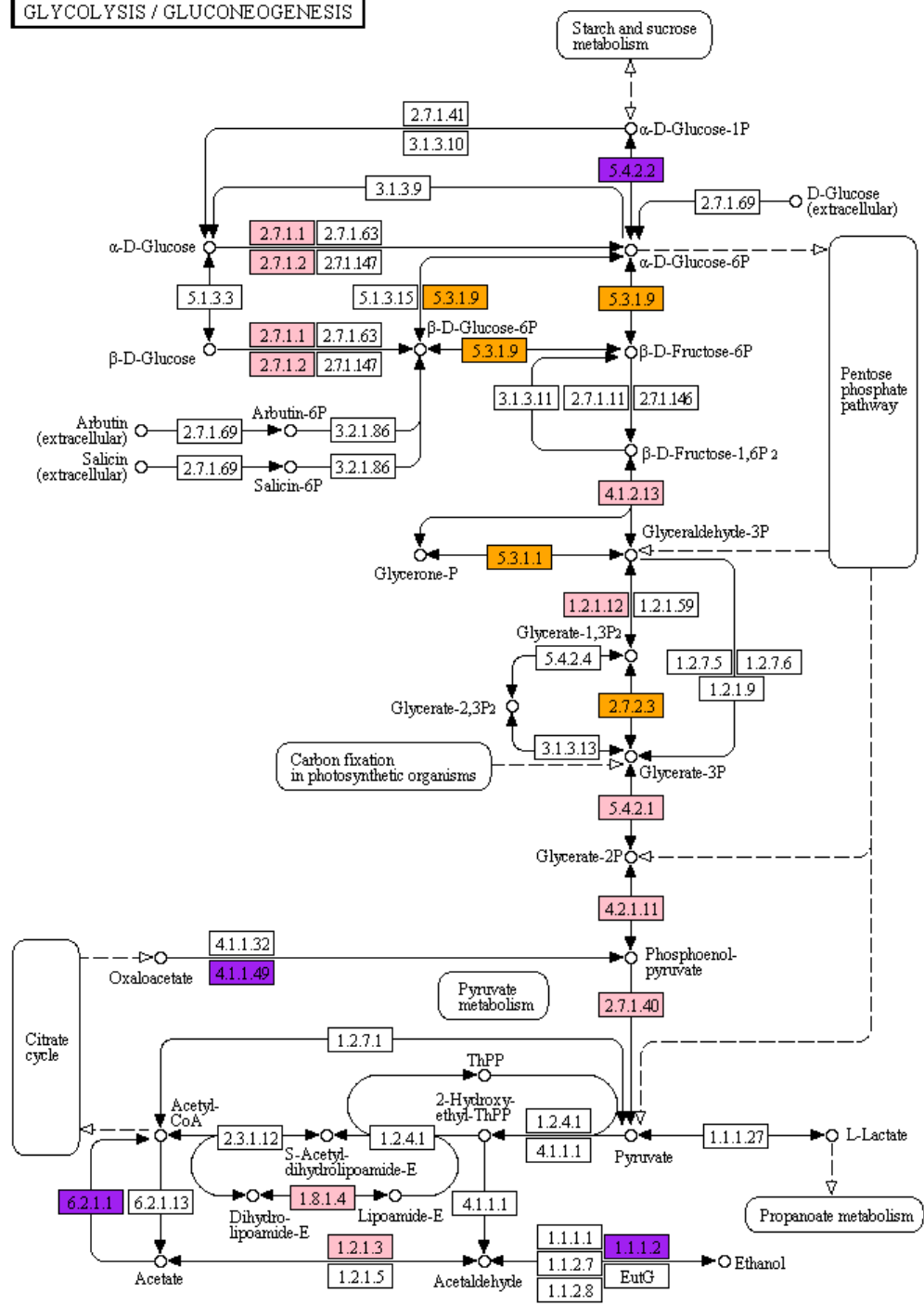

Supplement: Additional file 4: Figure S4 — Representation of glycolysis showing enzymes encoded by transcripts identified at 10 dai. Color coding as in Additional file 1: Figure S1, with the addition of boxes colored in pink representing enzymes encoded by both newly identified transcripts specific to 10 dai and transcripts sharing similarity to previously identified P. pachyrhizi ESTs and boxes colored in purple represent enzymes encoded only by transcripts sharing similarity to previously identified P. pachyrhizi ESTs. [file 1471-2164-14-614-S4.pdf]
